# Laparoscopic trocar management of a giant paraovarian cyst: a case report

**DOI:** 10.12688/f1000research.2-29.v2

**Published:** 2013-05-22

**Authors:** Mohamed Kandil, Tarek Sayyed, Mohamed Zakaria

**Affiliations:** 1 Department of Obstetrics and Gynecology, Faculty of Medicine, Menofyia University, Shibin El-kom, Egypt

**Keywords:** paraovarian cyst, laparoscopic trocar, adolescent

## Abstract

A 17-year-old woman had undergone exploratory laparotomy because of a huge cystic pelviabdominal mass equivalent of 36 weeks' gestation. A closed system drainage maneuver was applied via using a laparoscopic trocar to drain a revealed large left paraovarian cyst. This maneuver was found to be a simple and effective method to safely aspirate giant paraovarian cysts; thus allowing their total excision.

## Introduction

Paraovarian cysts occur in the broad ligament between the ovary and the tube, predominantly arising from mesothelium covering the peritoneum (mesothelial cyst) but also occasionally arise from the paramesonephric tissue (paramesonephric cysts or Mullerian cysts) and rarely from mesonephric remnants (mesonephric cysts or Wolffian cysts) ^1^. Paraovarian cysts constitute 10–20% of all adnexal masses ^2^. Some paraovarian cysts may reach a large size with possible complications like torsion and rupture ^3^. These cysts are usually benign and rarely malignant ^4, 5^. In this report, we present how we surgically managed a case with an abnormally huge paraovarian cyst.

## Case presentation

**Figure 1.**
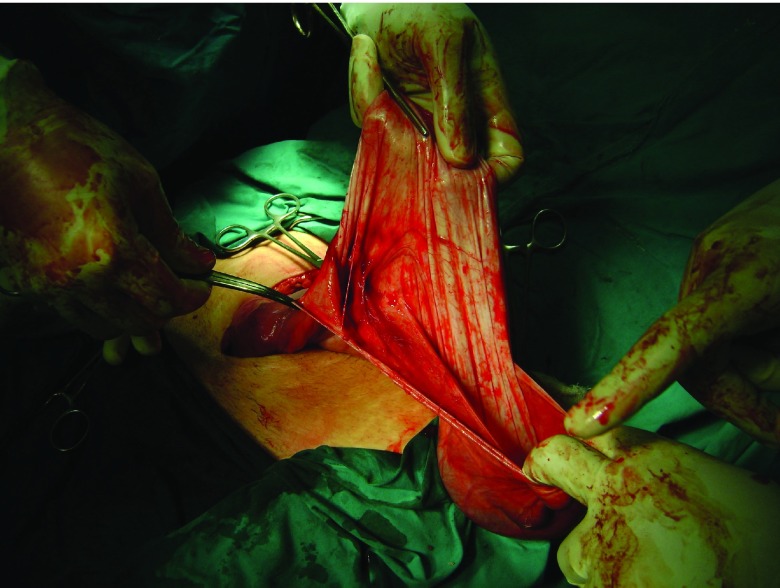
Appearance of the aspirated cyst wall after opening the broad ligament.

**Figure 2.**
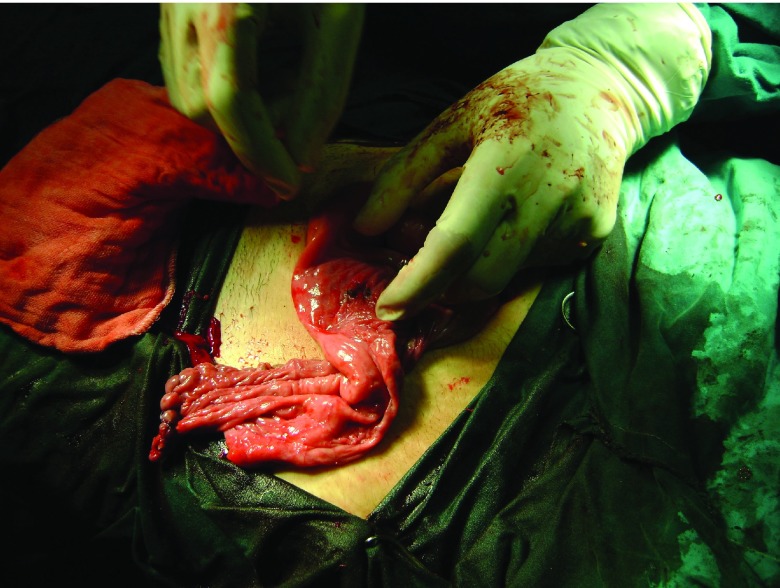
The final shape of the left broad ligament after its reconstruction with intact tube.

A 17 years old virgin presented with diffuse abdominal pain. History revealed a gradual increase of an abdominal swelling over the preceding 6 months. Physical examination showed a non tender, tense cystic pelvi-abdominal mass of 36 weeks gestational size. Computerised tomography revealed a 25 × 26 cm left ovarian simple cyst with clear contents and no septae. Serum CA125 level was normal. Other tumor markers were not performed due to financial constraint. Through a subumbilical midline incision, a huge smooth cystic mass overlying the whole abdominal cavity was found. The cyst was isolated from its surroundings with gauze packs. A loose purse string suture was placed in the lowest accessible part of the cyst. A 5 mm laparoscopic trocar with a side track off its main sleeve was connected to a high pressure suction irrigation device via a rubber tube. The trocar was then inserted through the center of the suture which was subsequently stretched to fit around the sleeve. This created a closed system to drain the cyst. The trocar was removed leaving its sleeve in place and suction drained eight liters of clear watery fluid. The collapsed cyst was found to be left paraovarian, which was exteriorized and the trocar sleeve was removed. The purse string suture was tightened to close the trocar opening. The left broad ligament was opened and the cyst wall was completely removed from the broad ligament ( Figure 1). The redundant peritoneum of the ligament was excised and subsequently reconstructed with preservation of the tubal integrity as seen in Figure 2. The patient had an uneventful postoperative recovery.

Postoperative histology reported simple benign serous cyst of mesothelial origin. The peritoneal fluid showed proteinaceous material entangling few lymphocytes and mesothelial cells with no evidence of malignancy.

## Discussion

Huge paraovarian cysts are uncommonly reported in the literature. We reviewed the English literature and were able to find three reports about the management of comparably huge cysts. In these reports, surgeons utilized abdominal incisions extending over the umbilicus, much larger than the one we used, in order to allow for cyst extraction and excision ^6– 8^. Only one report for three adolescents with large paraovarian cysts (with a range of 20–26 cm) had addressed decompression techniques before cyst exteriorization and excision using a suction cannula ^9^.

Two reports dealt with large paraovarian cysts laparoscopically. The first was a simple paraovarian cyst associated with pregnancy which measured 20 cm while the second with acute lower abdominal pain measured 12 cm ^10, 11^. Darwish et al. reported a series of smaller paraovarian cysts which had been excised laproscopically; the largest was not more than 13 cm ^12^. They reported either cyst aspiration and forceps coagulation of small cysts or cyst wall trocar puncture followed by suction and extraction for large cysts. Laparoscopy would have been technically difficult in our case due to the huge size of the cyst reaching close to xiphesternum. Direct abdominal entry with a Veress needle or trocar might have traumatized the cyst leading to spillage of its content. It is the authors’ view that laparoscopic cyst decompression before its removal is associated with greater risk of cyst spillage than the technique we describe. We therefore, through laparotomy and a small surgical incision, employed a closed drainage system to safely aspirate the cyst followed by excision.

## Conclusion

Open surgery remains the gold standard route to deal with giant paraovarian cysts. Aspiration of the cyst using a closed system followed by excision is a safe and effective treatment.

## Consent

Written informed consent for publication of the clinical details and clinical images was obtained from the patient.
