# Supplementary material for: Laparoscopic trocar management of a giant paraovarian cyst: a case report
Source: F1000Res. 2013 May 22;2:29. Originally published 2013 Jan 30. [Version 2] doi: 10.12688/f1000research.2-29.v2 (PMC3752714; doi:10.12688/f1000research.2-29.v2)
Supplement: Peer review history

# Peer review: Referee response for version 2

**DOI:** 10.5256/f1000research.1495.r3291

**Published:** 2014-01-23

**Authors:** Sian Jones

**Affiliations:** 1 Department of Obstetrics and Gynaecology, Bradford Teaching Hospitals NHS Foundation Trust, Bradford, UK

I have read this submission. I believe that I have an appropriate level of expertise to confirm that it is of an acceptable scientific standard.

# Peer review: Referee response for version 1

**DOI:** 10.5256/f1000research.1187.r913

**Published:** 2013-04-29

**Authors:** Daniel Kruschinski

**Affiliations:** 1 EndoGyn®, Endoscopic Gynecology Centers, Germany

This case is interesting and shows the ability to manage big ovarian cysts laparoscopically. Important issues during the procedure, like the closure of the cyst incision site with sutures to prevent spillage, were correctly addressed.

I have read this submission. I believe that I have an appropriate level of expertise to confirm that it is of an acceptable scientific standard.

# Peer review: Referee response for version 1

**DOI:** 10.5256/f1000research.1187.r794

**Published:** 2013-02-22

**Authors:** Sian Jones

**Affiliations:** 1 Department of Obstetrics and Gynaecology, Bradford Teaching Hospitals NHS Foundation Trust, Bradford, UK

An interesting case report that may help others when they come to deal with similar cases. A little more detail in the other methods alluded to in the laparoscopic cases might be helpful. The grammar and language needs editing.

I have read this submission. I believe that I have an appropriate level of expertise to confirm that it is of an acceptable scientific standard.
